# Effects of silibinin on hepatic warm ischemia-reperfusion injury in the rat model

**DOI:** 10.22038/ijbms.2019.34967.8313

**Published:** 2019-07

**Authors:** Vahid Akbari-Kordkheyli, Soheil Azizi, Abbas Khonakdar-Tarsi

**Affiliations:** 1 Student Research Committee, Mazandaran University of Medical Sciences, Sari, Iran; 2 Department of Laboratory Medicine, Faculty of Allied Medical Sciences, Mazandaran University of Medical Sciences, Sari, Iran; 3 Department of Biochemistry, Biophysics and Genetics, Cellular and molecular biology research center, Faculty of Medicine, Mazandaran University of Medical Sciences, Sari, Iran

**Keywords:** eNOS, ET-1, KLF2, Liver Ischemia/reperfusion, Silibinin

## Abstract

**Objective(s):**

Liver ischemia-reperfusion injuries (I/RI) are typically the main causes of liver dysfunction after various types of liver surgery especially liver transplantation. Radical components are the major causes of such direct injuries. We aimed to determine the beneficial effects of silibinin, a potent radical scavenger on liver I/RI.

**Materials and Methods:**

Thirty-two rats were divided into 4 groups. Group I: VEHICLE, the rats underwent laparotomy and received DMSO, group II: SILI, laparotomy was done and silibinin was administered. Group III: I/R, the rats received DMSO and were subjected to a liver I/R procedure and group IV: I/R+SILI, the animals underwent the I/R procedure and received silibinin. After 1 hr of ischemia followed by 3 hr reperfusion, blood was collected to evaluate the serum marker of liver injuries. Hepatic tissue was harvested to investigate glycogen content, histological changes, and vasoregulatory gene expression.

**Results:**

Results showed that serum AST, ALT, LDH, GGT, ALP, and hyaluronic acid (HA) increased significantly in I/R group compared with the VEHICLE group. Silibinin reduced this elevation except for GGT. Silibinin inhibited hepatocyte vacuolization and degeneration, endothelium damages, sinusoidal congestion and inflammation, and glycogen depletion during I/R. ET-1 mRNA was overproduced in the I/R group compared with the VEHICLE group which was decreased by silibinin. KLF2 and eNOS expression was reduced during I/R compared with the VEHICLE group. Silibinin elevated KLF2 expression but had no meaningful effect on eNOS expression.

**Conclusion:**

Silibinin protected the liver from I/RI. Silibinin could improve liver circulation by preventing sinusoidal congestion, inflammation, and perhaps modification of the vasoregulatory gene expression.

## Introduction

Ischemia-reperfusion (I/R) is a deleterious phenomenon occurring during a transient obstruction of tissue circulation (ischemia) followed by restoration of blood flow into it (reperfusion). The liver has a high vulnerability to ischemia-reperfusion injury (I/RI) owing to the fact that 75% of liver required-blood is provided by the portal vein and just 25% of remaining is supplied by the hepatic artery (rich in O_2_) (1). Liver I/R occurs under different clinical conditions, e.g., biopsy, tumor surgery, trauma, hemorrhage, sepsis, and most importantly in liver transplantation. Starvation, age, steatohepatitis, duration and severity of ischemia and reperfusion are the clinical factors affecting the intensity of I/R damages (2). Ischemia induces oxidative stress, inflammatory response, microcirculation impairments and subsequent cell death. During ischemia, the reduction in tissue oxygen and glucose supplies trigger cellular ATP reduction, changes in cellular membrane transitions, edema, and reactive oxygen species (ROS) production. ROS including superoxide radical anion, hydroxyl radical, singlet oxygen, and hydrogen peroxide are the mediators of oxidative stress that damage cellular targets such as DNA, proteins, and lipids. After an ischemia, reperfusion may lead to the consecutive chain of the pathophysiological cascades such as massive intracellular Ca^2+^ release, vasoconstriction, mitochondrial electron transport chain impairment, recruitment of neutrophils, acute inflammatory responses, and overproduction of ROS and reactive nitrogen species (RNS), which elevate apoptotic and necrotic cell death (3, 4). 

Microcirculation failures have a key role in I/RI. Intervention in vasoregulatory gene expression is a proper strategy to repair the failures. Among these genes, endothelin-1 (ET-1) and endothelial nitric oxide synthase (eNOS) are considered having the most importance. ET-1 which is overproduced by sinusoidal endothelial cells during I/R leads to venous contraction and inefficient reperfusion (5). eNOS and some other vasodilators such as TM and HO-1 are induced by kruppel like factor 2 (KLF2), a novel vasoprotective transcription factor that presents in the vascular endothelium. So, KLF2 derived atheroprotective pathways can have desirable effects on maintaining vascular tone during I/R (6).

The luminal surface of endothelium is covered by glycocalyx (GCX), which prevents inflammatory cell infiltration by making a barrier between them and the sinusoidal endothelial surface. HA, the most sensitive components of the GCX to reactive oxygen species (ROS), can be broken and released into the circulation following ROS invasion. These HA particles have the capacity of activating leukocytes that worsen I/RI. So, HA can be considered an appropriate indicator of I/RI (7).

It was reported that ROS overproduction in reperfused tissues is able to dysregulate signaling pathways of vasomotor gene expression directly or via increasing inflammatory mediators which lead to an imbalance of vasoconstrictor/vasodilator. Thus, radical scavengers can be protective against microcirculation injury during I/R (8). Silibinin, as flavonolignan, is the main component (70-80%) of silymarin (an extracted material from *milk thistle*). Efficacy of silymarin/silibinin on tissue ischemia and hepatocyte regeneration is well documented (9, 10). We designed this study to determine the possible mechanism of action of silibinin on liver warm I/R.

## Materials and Methods

For this experimental study, thirty-two male Wistar albino rats weighing 220±20 g were provided from the animal laboratory of Mazandaran University of Medical Sciences. The rats were kept in an air-conditioned room with a temperature of 23±2 °C and humidity of 55% ±5 under 12 hr dark/light cycles. Tap water and normal diet were *ad libitum*. The experimental protocol was approved by the animal ethical committee of MAZUMS. After two weeks of adaptation, the rats were randomly divided into four groups each containing 8 rats: group I, VEHICLE: each animal received DMSO intraperitoneally and underwent only laparotomy, group II, SILI: this group received silibinin and simultaneously underwent laparotomy, group III, I/R: the animals were subjected to I/R and received DMSO and group IV, I/R+SILI: the animals underwent I/R and received silibinin. All parameters in the studied groups were assessed in blinded fashion.

***Surgery procedure***

All of the surgical processes were performed under sterile conditions at 8-13 o’clock to prevent time-dependent varieties. The rats fasted for 18 hr before the beginning of surgery but tap water was freely accessible. Ketamine (50 mg/kg, IP) and Xylazine (8 mg/kg, IP) were injected for general anesthesia. Then midline laparotomy was done, and the liver was brought out to make the triad port (portal vein, hepatic artery, and biliary duct) visible. Subsequently, the left branch of triad port was obstructed by single metallic bulldog clamp to induce complete ischemia of the median and left lobes. The right and tail lobes remained perfused to prevent intestinal congestion. During ischemia, the liver was kept wet with a moist sterile gas to prevent dehydration and the rat was positioned under surgical lighting to maintain body temperature. After 1 hr ischemia, the clamp was carefully removed and the liver inserted into the abdominal cavity to allow reperfusion for 3 hr; the incision location was sutured. The VEHICLE and SILI groups were prepared in a similar way, but vascular clamping wasn’t applied.

***Silibinin administration***

**Figure 1:**
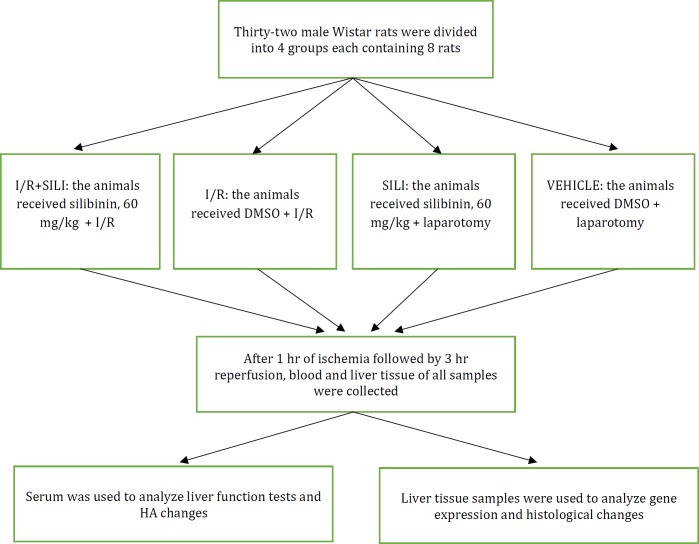
Schematic diagram of the study design. I/R: ischemia-reperfusion. HA: hyaluronic acid

Silibinin with about 98% purity was purchased from the Sigma Company (Sigma Chemical Co., St. Louis, MO, U.S.A.). Due to its poor solubility in aqueous solution (0.5 gr/l H_2O_), silibinin was dissolved in 10% DMSO (Merck) in normal saline 10 min before administration (11). Silibinin was administered twice intraperitoneally in a total dosage of 60 mg/kg, 30 min before laparotomy and immediately after the beginning of reperfusion, each time 30 mg/kg and a final volume of 0.5 ml (Figure 1).

***Biochemical*** analysis

After 3 hr of reperfusion, blood samples of all rats were collected from inferior vena cava under complete anesthesia. Serum was separated via centrifuging at 3000 rpm for 10 min and stored at 70 ^°^C until initiation of biochemical experiments. The activities (U/l) of serum aspartate aminotransferase (AST), alanine aminotransferase (ALT), γ-glutamyl transferase (GGT), alkaline phosphatase (12), lactate dehydrogenase **(**LDH), and the total bilirubin (T.Bil) levels (μmol/l) of all samples were evaluated spectrophotometrically with the Pars azmoon kit (Karaj, Iran) using a biochemical autoanalyzer (BT-3000-plus, Biotechnica, Italy). Serum HA was determined (ng/ml) by a rat HA ELISA kit (R&D systems^TM^ a bio-techno brand, USA).

***Histological examination***

Simultaneously with blood sampling, liver sections were harvested from the ischemic lobes of all rats. The tissues were fixed in 10% PBS-buffered formalin for hematoxylin and eosin (H&E) staining and 10% neutral-buffered formalin for periodic acid Schiff (PAS) staining. To prepare specimens for microscopic investigation, all tissue samples were washed with water to remove fixative liquid, and dehydrated with different grades of alcohol (50-100%), then cleaned with Xylene, and finally filled with paraffin. Sections, about 3–5 micrometer thickness, were cut with a microtome, deparaffinization was done with xylene. Lastly, the tissue was stained with H&E and its glycogen content visualized by staining with PAS. Histological examination was performed by an experienced pathologist in blinded fashion according to the modified scoring system and the severity of the lesions taken from previous studies. Each parameter was scored between NO or few changes (<10%), mild changes (10-30%), moderate changes (31-50%), and marked changes (>50%) compared to the VEHICLE group.

***Total RNA extraction and reverse transcriptase-polymerase chain***

*Reaction (RT-PCR)*

Total RNA of all specimens was isolated from 30 mg of liver sections (had been kept in RNA later (Sigma) in -70^ °^C) according to RNeasy plus mini kit (Qiagen, Ger) protocol. RNA Purity was verified by spectrophotometric absorbance ratio in 260/280. Also, the RNAs were resolved by electrophoresis in agarose gel 1% stained with SYBR Green for about 40 min in 80V and the results were checked by possessing two sharp bands for 18S and 28S ribosomal RNA. cDNA was synthesized from 5 μg of the RNA in just 30 min according to QuantiTect RT-PCR kit (Qiagen, Ger) protocol. Real-time PCR Step-One Plus^TM^ reactions were performed with Rotor- Gene^®^Q in total volume of 25 µl: 12.5 µl SYBR Green PCR Master Mix reagent (Qiagen, Ger), 2 µl cDNA, 0.5 µl R and F primers for β-actin and ET-1, and 1.5 µl mixed R and F primer for KLF2 (final concentration of primers was 10 pM) and DDwater up to 25 µl. Sequence of primers was as follows: ET-1, 5′- AGC ACA TTG ACT ACA GAC C-3′ (F), 5′-ACG AAG ACA GGT TAG GGA A-3′ (R), eNOS, 5′-GGA TCC AGT GGG GGA AAC TG-3′(F), 5′-TGG CTG AAC GAA GAT TGC CT3′ (R), and β-actin as housekeeper gene, 5′-CCC ATC TAT GAG GGT TAC GC-3′ (F), 5′-TTT AAT GTC ACG CAC GAT TTC-3′ (R). The sequence of KLF2 primers designed by Qiagen was not reported. Lengths of PCR products for ET-1, β-actin, KLF2 and eNOS were 212, 149, 150, and 123 base pairs (bp), respectively. All experiments were carried out duplicated following this protocol: 15 min in 95 ^°^C (to activate Taq polymerase hot start) and 40 cycle: 20 Sec at 95 ^°^C (denaturing), 15 Sec at 60 ^°^C (annealing) for β-actin and ET-1, but 57 ^°^C for KLF2 and eNOS, and 15 min at 72 ^°^C (extension) for all genes. Annealing temperatures were determined with a temperature gradient by normal PCR. 10 µl of amplified products were resolved by electrophoresis in agarose gel 2% stained with SYBR Green for 40 min in 80V to confirm quality and specialty of PCR products. Melting curve for each sample was surveyed of having just a single peak.

***Statistical analysis***

AST, ALT, LDH, ALP, GGT, HA, and T.Bil were expressed as mean±standard error of the mean (mean±SEM). The mean difference between groups was determined by one-way ANOVA and Tukey’s multiple comparison tests using the SPSS 18 software package; the level of *P*<0.05 was considered significant. Real-time PCR results were analyzed using the following formula:

RQ =2^-ΔΔCT^, where Ct = Ct (test) – Ct (calibrator)

***Biochemical results***

As shown in Table 1, there were no statistical differences in serum AST, ALT, GGT, ALP, LDH, HA, and T. Bil between VEHICLE and SILI groups. But in the I/R group, these markers except T.Bil were elevated markedly compared to VEHICLE groups. In the I/R+SILI group, silibinin treatment could significantly attenuate these elevations except GGT, but not the scales of the VEHICLE group.

***Histological results***

**Figure 2:**
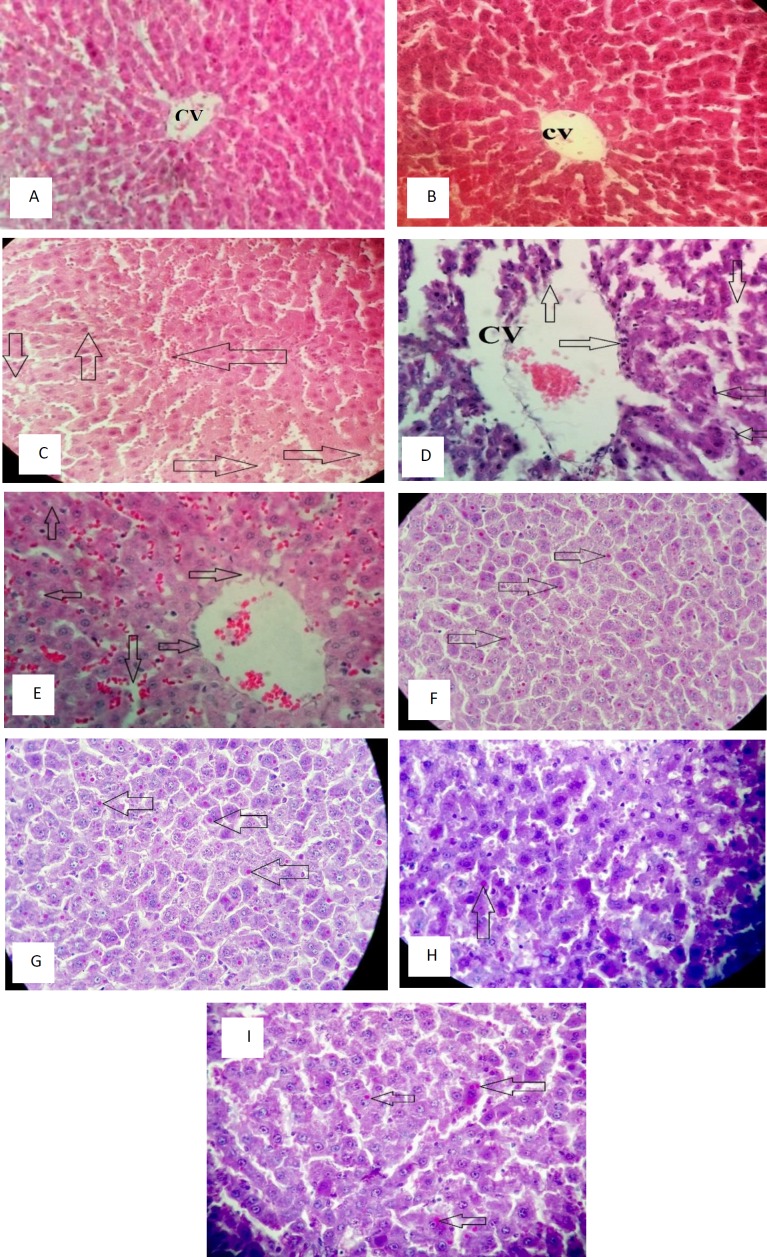
Vehicle group. Normal structure and radial alignment around the central vein (CV) (A); SILI group, health CV endothelium (B); I/R group, down and up arrows: cytoplasmic vacuolization. Right arrows: hydrotropic degeneration. Left arrow: sinusoidal congestion (C); I/R group. Up arrow: separation of sinusoidal endothelial cells and enlargement of CV. Right arrow: neutrophil infiltration around CV. Down arrow: dilated sinusoids. Left arrows: activated and marginated Kupffer cells (D); I/R+SILI group. Up arrow: mild degeneration, left arrow: minimal vacuolization, right arrows: less damaged CV endothelium, and down arrow: limited dilation (E); Vehicle group. Arrows: glycogen deposition in hepatocyte cytoplasm (F); SILI group. Homogenous distribution of glycogen (G); I/R group. The large area of glycogen depletion (H); I/R+SILI group. Less frequent glycogen depletion (I)

Histological evaluation revealed that the vehicle and silibinin groups had a normal structure of liver tissue (Figures 2A, B). But in the I/R group, liver parenchyma was significantly damaged. Sinusoids were congested and dilated. Kupffer cells were activated moderately. Neutrophil infiltration and recruitment were observed in sinusoids and around the central vein of lobules. There were hydropic degeneration and cytoplasmic vacuolization in several instances, but necrosis was scarcely seen in zone 3 of liver tissue (Figures 2C, D). In I/R+SILI group, liver cells showed less injured morphology. Activated Kupffer cells and neutrophil infiltration were mild. Sinusoidal endothelium was less damaged. Cellular degeneration and vocalization were seen just in a few cases and hepatocyte necrosis wasn’t observed at all (Figure 2E). Histological alterations are summarized in Table 2. Results of PAS staining demonstrated that hepatocyte glycogen storage was decreased markedly in I/R group compared to silibinin treatment I/R group (Figures 2F-I). 

***Real-time PCR results***

**Figure 3:**
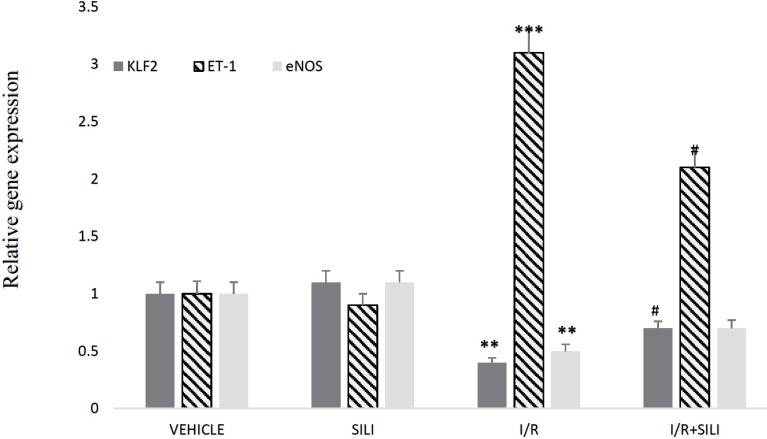
Relative KLF2, ET-1 and eNOS gene expression between the four studied groups. ** (*P*<0.01) and *** (*P*<0.001) compared to VEHICLE group. # (*P*<0.05) compared to I/R group. KLF2: Kruppel-like factor 2; ET-1: endothelin-1; eNOS: endothelial nitric oxide synthase

Analysis of real-time PCR results showed that the mRNA levels of KLF2, eNOS, and ET-1 statistically had no differences in the VEHICLE and SILI groups, but in I/R group KLF2 and eNOS gene expression decreased significantly compared to VEHICLE group. In I/R+SILI group, silibinin could improve the KLF2 expression compared to I/R group but had no meaningful effect on eNOS expression. Although ET-1 mRNA level enhanced markedly in I/R group compared to the control group, that was inhibited by silibinin in I/R+SILI group (Figure 3).

## Discussion

I/RI are the cause of about 80% of liver dysfunction after transplantation procedures (13). The pathogenesis of I/RI is multifactorial, complex and involves ATP depletion, hepatocyte edema, acidosis, oxidative stress, inflammatory responses, liver cell death, and microcirculation defects. These events may progress to systemic inflammatory response syndrome and multiple organ dysfunction syndromes (a consequence of toxic metabolites effusion via the systemic circulation) and eventually graft rejection (14, 15). Microcirculation injuries marked as the critical determinants in I/R lesions, in this regard, physically preventing circulation by means of flow-controlled reperfusion, mainly inhibited isolated and perfused liver parenchymal cells from necrosis after I/R (16). Some studies reported that attenuating of liver microcirculatory injuries depended on antioxidant exposure. Since liver usual antioxidants are decreased due to rising of ROS during I/R, antioxidant therapy could be an appropriate strategy to reinforce microcirculation in this status (17, 18). Silibinin is a blend of silybin A and B (ratio 1/1) that constitute 80% of the silymarin component, so have the main role in silymarin beneficial effects. In addition to ROS scavenging, silibinin/silymarin decreased TNF-α, PGE2, and IL-1β via inhibiting NF-κB expression in liver suffering inflammation (9, 19-21). We evaluated the effects of silibinin on liver parenchyma injury and vasoregulatory gene expression in hepatic warm I/R. Our study indicated that silibinin could decrease serum levels of AST, ALT, LDH, and ALP during I/R. These markers are released from the ROS-destroyed plasma membrane. Some studies showed that the serum level of these markers raised during reperfusion with a maximal peak at 12 hr after reperfusion (22). It seems that silibinin can stabilize cell membranes by neutralizing large scales of ROS that lead to a decrease in the serum marker of liver injuries during I/R. 

Our histological examination revealed that silibinin could ameliorate liver histological damages during I/R. In addition to ATP depletion, edema, and intracellular source of ROS, inflammatory responses have a major role in histological lesions. DAMPs releasing from injured cells lead to Kupffer cell activation during ischemia (acute phase). These cells produce ROS (extracellular source) and cytokines such as TNF-α and IL-1 that trigger to neutrophil and another inflammatory cell chemotaxis (lateral phase); these cells produce more ROS, RNS, and cytokines like IL-8 that result in more activities of Kupffer cells. So, inflammatory cells have synergistic effects on each other (23). These mounts of radical components cause hepatocytes degeneration and vocalization, sinusoidal and venous endothelial damages, and leukocyte infiltration in liver tissue as shown in the microscopic investigation. We observed Silibinin could attenuate the histological injuries. Its protective effects may be due to reducing inflammatory factors, increasing protein synthesis and hepatocyte regeneration that were reported in a number of studies (24). Also, we determined that massive depletion of hepatocyte glycogen storage during I/R was prevented by silibinin. In addition to a marked increment of glycolysis during I/R, cellular and membrane degradation could result in loss of glycogen.

It has been illustrated in many studies that the vasomotor gene expression is dysregulated during I/R. It seems that alteration in the blood mechanical forces on the endothelial surface can be transduced to intracellular signaling pathways by mechanotransduction complexes that can lead to flow-specific gene expression. Eventually, these events result in an imbalance between the ratio of vasodilators and vasoconstrictors specially eNOS/ET-1, as the most important of them (5). ET-1 peptide is capable of reversible contraction of liver sinusoids via increasing of Ca^2+ ^influx into cells, particularly stellate cells. Our results showed that ET-1 mRNA level was definitely raised pending I/R in line with other studies (25). It was proven that increasing of ET-1 results in the no-reflow phenomenon, portal and venous hypertension and congestion, leukocyte adhesion, and eventually microcirculation resistance (26). Other studies revealed that inhibition of ET-1 by a monoclonal antibody or suppressing its receptors, protected the liver from warm I/R (27). The exact mechanism of increased ET expression in I/R has not been understood yet. But, in our opinion, endothelial cells that are directly exposed to blood dynamic can trigger vasoconstriction as a physiological response to low blood flow during ischemia. Under these conditions, endothelium tries to maintain homeostatic blood pressure via releasing of vasoconstrictors such as TXA_2_, ICAM and in particular ET-1 and decreasing vasodilators such as NO that was proven in some studies (26). Our findings showed that silibinin reduced ET-1 mRNA in I/R status that was accompanied with less leukocyte adhesion and congestion in histological examination.

It was well documented that KLF2 had the capacity to stimulate the expression of the vasoprotective genes such as HO-1, eNOS, and TM (28). In addition to the vasodilatory effect, eNOS derived NO, can inhibit the expression of vascular adhesion molecules (29). Therefore, KLF2 contributes to reducing microcirculatory injuries by up-regulation of the atheroprotective genes program. Our real-time PCR results exhibited that the level of KLF2 mRNA decreased pending I/R that was in harmony with reducing eNOS expression. The possible causes of I/R-induced reduction in KLF2 expression are different. Some researchers identified that the extracellular ATP is an important inducer of KLF2 expression, whereby using an apyrase (an ATPase) could reduce the levels of KLF2 mRNA in shear stress condition (30, 31). Also, a recent study showed that some inflammatory cytokines such as TNF-α, IL-1β, and NF-κB are able to reduce KLF2 expression; in this study, reduction of these cytokines led to increasing of KLF2 mRNA (32). Therefore, the elevation of inflammatory cytokines and reduction in ATP may be the two probable causes of decreased KLF2 mRNA during I/R. Marrone *et al.* concluded that the statins promoted KLF2 expression in the rat cirrhotic liver was accompanied by a marked increase in eNOS and TM levels (33). Gracia-Sancho *et al.* found that resveratrol was able to stimulate the expression of KLF2, eNOS, TM, and CNP in cultured endothelial cells. Also, they determined that Resveratrol-induced eNOS, TM, and CNP expression was dependent on KLF2 expression (34). Our results characterized that silibinin was able to stimulate KLF2 expression in I/R condition but had no meaningful effect on eNOS mRNA level. Also, in our previous study, silibinin at a dose of 100 mg/kg had no effect on eNOS expression in rat liver insulted by I/R (3 hr of ischemia followed by 5 hr of reperfusion) (35).

**Table 1:** Serum biochemical markers of liver function in four studied groups

**Group**	**AST**	**ALT**	**LDH**	**GGT**	**ALP**	**T.Bil.**	**HA**
**VEHICLE**		125.2±10.1	65.5±5.23	453.3±36.1	8.1±0.7	171.9±14.3	1.6±0.1	5.2±0.4
**SILI**	122.3±10	61.9±5.19	449.7±35.9	8±0.7	169.8±14.1	1.5±0.1	5.1±0.4
**I/R**	610.8±50.3^***^	751.6±53.7^***^	1721.9±150.7^***^	12.4±0.9^*^	341.5±28.9^*^	1.8±0.1	20.9±1.5^***^
**SILI+I/R**	251.3±18.7^###^	252.4±17.3^###^	650.8±48.1^###^	10.2±1.1	250.4±23.5^#^	1.7±0.1	10.6±0.8^###^

The values were expressed by mean±SEM of 8 rats per group. The level of significance was considered as *P*<0.05. *** and * show significant differences compared to the VEHICLE group with the values of *P*<0.001 and *P*<0.05, respectively. ### and # display significant differences compared to the I/R group with the values of *P*<0.001 and *P*<0.05, respectively. AST: aspartate aminotransferase; ALT: alanine aminotransferase; LDH: lactate dehydrogenase; GGT: γ-glutamyl transferase; ALP: Alkaline Phosphatase; T.Bil.: total bilirubin; HA: hyaluronic acid

**Table 2:** Comparison of histological injuries in four studied groups

	VEHICLE	SILI	I/R	I/R+SILI
**Distortion radial alignment around the** **central vein**	-	<10%	31-50%	10-30%
**Decreased cellularity**	-	<10%	<10%	<10%
**Central vein and sinusoidal congestion **	-	<10%	31-50%	10-30%
**Sinusoidal dilatation**	-	<10%	31-50%	10-30%
**Neutrophil infiltration **	-	<10%	31-50%	10-30%
**Hepatocyte necrosis**	-	<10%	10-30%	<10%
**Hydropic degeneration**	-	<10%	31-50%	10-30%
**Activated Kupffer cells**	-	<10%	31-50%	10-30%
**Cytoplasmic vacuolization**	-	<10%	31-50%	10-30%
**Eosinophil infiltration**	-	<10%	<10%	<10%
**Endothelial injury**	-	<10%	31-50%	10-30%

<10% NO or few changes, 10-30% mild changes, 31-50% moderate changes comparison to Vehicle group

GCX is a critical factor in endothelial permeability (36). HA, a ROS-sensitive constituent of GCX is fragmented to particles sized 1-30 kDa by ROS pending I/R. HA particles are able to intensify leukocyte activation (7). In addition, changes in the endothelium phenotype can trigger the flow cessation. During I/R, large amounts of HA particles can be released which would be much higher than the macrophages and endothelial cells (main source of HA particles) capacity to uptake and catabolize them (37). In some studies, serum elevated HA was evaluated as an injury marker of liver fibrosis and muscle I/R (38, 39). In the healthy rat, the destruction of HA with hyaluronidase could induce endothelial GCX injuries in rat cremaster microcirculation as I/R could induce (40). Other investigations reported that the injection of exogenous hyaluronan or allopurinol (XOD inhibitor) could improve I/R induced sinusoidal endothelial GCX damages (39). In this study, we determined that silibinin could lessen the notably I/R increased serum HA level. It seems that silibinin prevents HA fragmentation by neutralizing ROS produced during reperfusion.

## Conclusion

In this study, we determined that the post/pre-operative injection of silibinin could protect the liver from I/RI. Silibinin could improve liver circulation by preventing congestion, inflammation, HA release, and perhaps rectifying vasoregulatory gene expression. Silibinin maintained liver tissue structure and glycogen storage during I/R. 
